# Survival and risk factors associated with mortality in people living with HIV from 2005 to 2018 in Nanjing, China

**DOI:** 10.3389/fpubh.2022.989127

**Published:** 2022-10-19

**Authors:** Zhengping Zhu, Yuanyuan Xu, Sushu Wu, Xin Li, Hongjie Shi, Xiaoxiao Dong, Wenjiong Xu

**Affiliations:** ^1^Department of AIDS and STDs Control and Prevention, Nanjing Municipal Center for Disease Control and Prevention, Nanjing, China; ^2^Department of Microbiological Test, Nanjing Municipal Center for Disease Control and Prevention, Nanjing, China

**Keywords:** HIV, AIDS, survival, mortality, treatment

## Abstract

**Background:**

Although the introduction of antiretroviral therapy (ART) decreased the mortality of people living with Human Immunodeficiency Virus (PLHIV), substantially, hundreds of thousands of people are dying of AIDS each year. The accurate survival patterns and factors related to death among PLHIV were rarely reported. In this study, we evaluated survival status and identified factors associated with death among PLHIV in Nanjing.

**Methods:**

We conducted a retrospective analysis of PLHIV followed-up in Nanjing and registered to the national HIV/AIDS comprehensive management information system from 2005 to 2018. We used the life table to calculate the cumulative survival rates. We applied the Kaplan-Meier to calculate median survival times and employed cox hazard proportional regression to analyze the associated factors related to death.

**Results:**

The median survival time of PLHIV was 11.8 (95%*CI*:11.6–11.9) years from 2005 to 2018. Among 4,235 PLHIV included in this study, 7.5% had died of AIDS-related disease and the AIDS-related mortality rate was 2.0/100 PYs. The cumulative proportion surviving at the end of the interval was 95.2% over the 1st year, 94.0% over the 2nd year, 91.8% over the 5th year, and 85.4% over the 10th year, respectively. PLHIV who unaccepted ART showed a greater risk of death compared to those who accepted ART (AHR = 16.2, 95%*CI*:11.9~22.2). For baseline CD4 count, compared to CD4 < 200 cell/μL, higher CD4 count was demonstrated as a protective factor, with *AHR* = 0.2 (95%*CI*: 0.1~0.3) for ≥500 cell/μL, *AHR* = 0.3 (95%*CI*:0.2~0.4) for 350~499 cell/μL, *AHR* = 0.4 (95%*CI*:0.3~0.6 for 200~349 cell/μL). In addition, we observed a higher death risk in PLHIV who were screened through outpatient (*AHR* = 1.6, 95%*CI*: 1.1~2.2) and inpatient (*AHR* = 1.6, 95%CI: 1.1~2.5) compared to through VCT; the age of diagnosis was ≥50 years old (*AHR* = 9.5, 95%*CI*: 3.7~24.1) and 25~49 years old (*AHR* = 5.0, 95%*CI*: 2.0~12.3) compared to ≤ 24 years old; educated from junior and below (*AHR* = 3.4, 95%*CI*: 2.3~5.1) and Senior high school (*AHR* = 1.7, 95%*CI*: 1.1~2.7) compared to college and above.

**Conclusion:**

The AIDS-related mortality among PLHIV in Nanjing was relatively low. A higher risk for AIDS-related deaths were observed among PLHIV who unaccepted ART, whose baseline CD4 cell count was<200 cell/μL, older age, and lower educated.

## Introduction

After the introduction of combined antiretroviral therapy in 1996, the mortality of people living with Human Immunodeficiency Virus (PLHIV) decreased substantially (1, 2). Despite this progress, hundreds of thousands of people are dying of Acquired Immunodeficiency Syndrome (AIDS) each year. The global number of PLHIV (all ages) deaths from AIDS-related diseases has been estimated to be 690,000 [500,000–970,000] in 2019 (3).

In 2003, the Chinese government announced the “Four Frees and One Care” policy to ensure that PLHIV in poor urban and all rural areas have access to Antiretroviral Treatment (ART) (4). In 2010, the State Council issued the “five expansion and six strengthening” policy to promote HIV testing, early diagnosis, and timely implementation of standardized ART. With the standardized implementation of a series of measures, HIV-infected people have maintained a high survival rate for a long time (5). Even though, by the end of 2019, there were still 316,477 AIDS-related deaths reported in China (6).

After the first imported case and the first local case were reported in 1991 and 1994, respectively, AIDS became a tremendous challenge to public health management departments in Nanjing. In responding to the AIDS epidemic, Nanjing has implemented a series of comprehensive prevention and control measures. It began to carry out free ART for AIDS patients in 2003, and afterwards, the inclusion criteria of free ART have been continuously adjusted and broadened. Under the UNAIDS 90-90-90 target to help end the AIDS epidemic (7), Nanjing has provided free ART services for all PLHIV since 2016. However, some PLHIV remain unconvinced about the positive treatment effects and refuse to accept ART. For instance, the untreated rate of PLHIV in Nanjing was still close to 10% by the end of 2019 (8).

Some studies have reported survival status and mortality among HIV/AIDS patients, but the discrepancy still exists. For example, patients' cumulative proportions of survival vary greatly in different regions (9, 10). The hazard of AIDS deaths is higher in men compared with women and in heterosexual men compared with men who have sex with men (11). Furthermore, the patient's diagnosis timing, CD4 count level, and income also may influence death outcomes (12). It is of increasing significance for the accurate description of survival among PLHIV.

We have previously studied the survival analysis of patients who have accepted or are accepting ART (13, 14). However, we have not comprehensively studied the survival status of all PLHIV, as well as the impact of specific demographic characteristics and diagnosis background on death risk. Consequently, we conducted this study to evaluate the survival status and explore the associated factors to mortality among all PLHIV in Nanjing.

## Materials and methods

### Study design and data collection

A retrospective cohort study was conducted to collect data from the National HIV/AIDS Comprehensive Management Information System and ART Management System. The systems include PLHIV diagnosis information (screening source, laboratory confirmation date, diagnosis physician, and report institution), demographic characteristics, CD4+ T lymphocyte cell count (hereinafter abbreviate for “CD4 count”) records, and the date of follow-up and death.

People confirmed to get infected with HIV and followed up in Nanjing from January 01, 2005 to December 31, 2018 were included in this study. Only cases under the age of 15 were excluded. Each case had an automatically generated ID number in the system, and the ID number remained unchanged regardless of the transfer. Therefore, there was no duplicate case. As this study was retrospective, data analysis of HIV/AIDS surveillance in China, the right and the welfare of the subject were adequately protected; the National Center for AIDS/STD Control and Prevention considered that it could waive informed consent from the subjects.

The confirm date was defined as the start date of each PLHIV, and December 31, 2018 was designated as the deadline date. If the patient died from AIDS-related causes, the date of death was recorded as the outcome. If the patient died from other diseases, such as suicide, traffic accident, illegal drug overdose, etc., the case was censored on the last recorded date. If the death was not out of the hospital or reported by a relative, the date of death was confirmed from the patient's hospital records or from the National Cause of Death Surveillance System. If the patient survived through the deadline, namely, December 31, 2018, then it was the date of censorship. If the patient was lost to follow-up, the end date was assumed as the last follow-up date adding 45 days, which was half of the follow-up interval. The baseline CD4 count in this study was obtained from their follow-up records in the National HIV/AIDS Comprehensive Management Information System.

### Variables definition

Screening way: VCT referred to Voluntary Counseling and Testing conducted by working staff in CDC or Community-based Organization (CBO). Physical exams included physical examination, pregnancy/prenatal screening, blood donation test, and mandatory test in detention facilities. Outpatient included HIV test initiated by general practitioners or dermatologists or STD clinic physicians. Inpatient included in-hospital and preoperative HIV test. Patients who accepted ART included those who have accepted ART once or always, and patients who unaccepted ART included those who had never accepted ART. The baseline CD4 count referred to the first CD4 test result within 1 year after being diagnosed with HIV infection. If the patient had two or more CD4 count records during the time, we selected the first one, and if the patient had not any CD4 count record within 1 year of diagnosis, we included the patient in the undetected group.

### Statistical analyses

We defined mortality as the number of deaths per 100 person years (PYs). The numerator was the number of AIDS-related death; the denominator was the total number of observed years. Means, standard deviations, and constituent ratios were used to summarize continuous and categorical variables. Kaplan-Meier estimator was applied to calculate the median survival time of patients according to different categories. Moreover, the log-rank test of Kaplan-Meier was employed to examine median survival times by different categories. Univariate factors with a *P* < 0.1 were included in the Cox proportional hazard models, which were used to estimate the mortality hazard ratios among confounding factor levels. Adjusted hazard ratios (*AHR*s) with their 95% confidence interval (*CI*) were reported. All the tests were two-tailed, and the type 1 error rate was set to 5%. SPSS software was used for data analysis (version 20; IBM, Armonk, NY, USA).

## Results

### Survival status and survival analysis of PLHIV

A total of 4,242 PLHIV were confirmed and followed up in Nanjing from 2005 to 2018. After excluding seven cases under 15 years of age, 4,235 cases were employed for analysis in this study. By the end of 2018, a total of 90.8% of PLHIV survived, 7.5% had died of AIDS-related diseases, 0.5% died of suicide, traffic accident or other diseases, and 1.2 % were lost to follow-up. The overall observation time was 15,803.5 PYs. The AIDS-related mortality was 2.0/100 PYs. Among all AIDS-related death, 50.9% died within 6 months after diagnosis. The median survival time was 11.8 (95%CI: 11.6~11.9) years during the study period.

**Figure 1:**
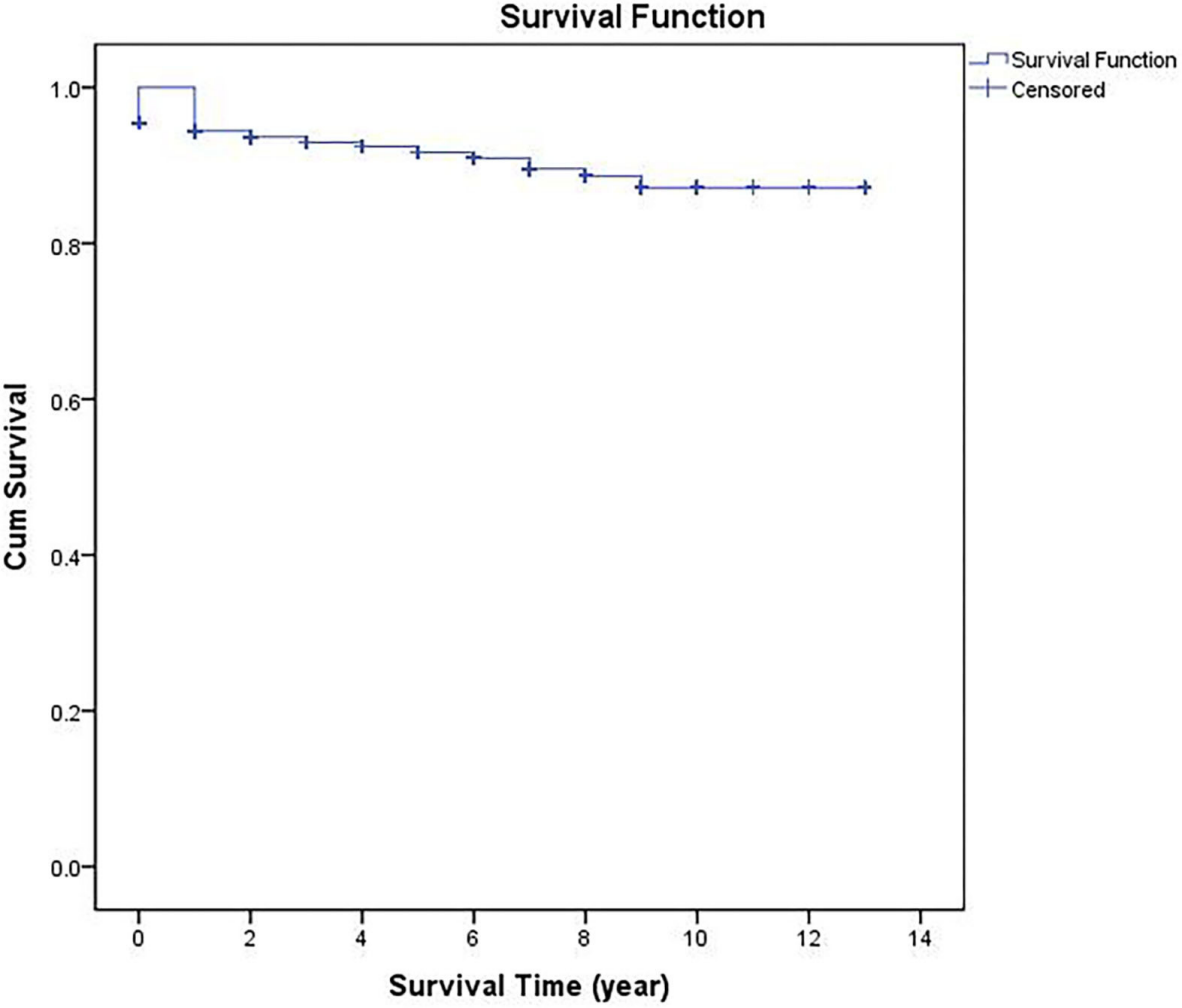
Survival curve for PLHIV from 2005 to 2018 in Nanjing, China.

According to the life table, the cumulative proportions surviving at the end of the interval were 95.2% in the 1st year, 94.0% in the 2nd year, 91.8% in the 5th year, and 85.4% in the 10th year (Table 1). As can be seen from the cumulative survival curve, PLHIV survival declined rapidly during the first year and gradually thereafter then reached its minimum value (85.4% survival) at the 10th follow-up year (Figure 1).

**Table 1:** Survival analysis of PLHIV from to 2005 to 2018 in Nanjing.

**Interval start time, yr**	**Number entering interval**	**Number withdrawing during interva**	**Number exposed to risk**	**Number of terminal events**	**Proportion terminating, %**	**Proportion surviving, %**	**Cumulative proportion surviving at end of interval, %**	**Std. error of cumulative proportion surviving at end of interval**
0-	4,235	518	3,976.0	192	4.8	95.2	95.2	0.00
1-	3,525	526	3,262.0	39	1.2	98.8	94.0	0.00
2-	2,960	536	2,692.0	24	0.9	99.1	93.2	0.00
3-	2,400	559	2,120.5	18	0.9	99.2	92.4	0.00
4-	1,823	503	1,571.5	10	0.6	99.4	91.8	0.00
5-	1,310	391	1,114.5	11	1.0	99.0	90.9	0.01
6-	908	317	749.5	7	0.9	99.1	90.1	0.01
7-	584	182	493.0	9	1.8	98.2	88.4	0.01
8-	393	154	316.0	4	1.3	98.7	87.3	0.01
9-	235	98	186.0	4	2.2	97.9	85.4	0.01
10-	133	63	101.5	0	0.0	100.0	85.4	0.01
11-	70	26	57.0	0	0.0	100.0	85.4	0.01
12-	44	27	30.5	0	0.0	100.0	85.4	0.01
13-	17	17	8.5	0	0.00	100.0	85.4	0.01

### Demographic characteristics of PLHIV

The mean age of 4,235 PLHIV was 35.2 ± 13.1 years, ranging from 15 to 91 years. Male accounted for 92.2%, single was 54.2%, and Han ethnic was 97.8%. In terms of educational level, 49.8% were college and above, 24.7% were senior, and others were junior and below. As to transmission ways, 69.0% were infected *via* homosexual contact, 25.7% were *via* heterosexual contact, 3.7% *via* injection drugs, and 1.7% were unclear. Among 4,235 PLHIV, 86.0% accepted ART. A total of 4,095 (96.7%) had baseline CD4 count records. In 4,095 cases who had baseline CD4 count record, <200 cell/μL group constituted 23.8%, 200–349 cell/μL group was 25.8%, 350–499 cell/μL was 25.4%, and ≥500 cell/μL 21.7% (see Table 2).

**Table 2:** Survival time and log-rank test of Kaplan-Meier among PLHIV in Nanjing.

**Variables**	**Patients *n* (%)**	**Deaths n**	**Median survival time, Yrs. (95% CI)**	**χ^2^**	***P* value**
Age of diagnosis				204.733	<0.001
15–24	991 (23.4)	8	12.9 (12.7–13.0)		
25–49	2,542 (60.0)	181	11.8 (11.6–12.0)		
≥50	702 (16.6)	129	10.2 (9.8–10.7)		
Gender				17.481	<0.001
Male	3,906 (92.2)	273	11.9 (11.7–12.0)		
Female	329 (7.8)	45	10.9 (10.0–11.5)		
Current marital status				119.773	<0.001
Single(never married)	2,294 (54.2)	76	12.3 (12.1–12.5)		
Married	1,283 (30.3)	147	11.3 (11.0–11.6)		
Divorced/widowed	618 (14.6)	85	11.1 (10.7–11. 5)		
Unknown	40 (0.9)	10	9.2 (7.6–10.7)		
Ethnic group				0.0360	0.850
Han	4,142 (97.8)	312	11.8 (11.7–11.9)		
Other minorities	93 (2.2)	6	10.0 (9.2–10.8)		
Screening way				133.058	<0.001
VCT	2,266 (53.5)	85	12.3 (12.1–12.5)		
Health exam	348 (8.2)	21	12.0 (11.6–12.4)		
Outpatient	1,185 (28.0)	146	11.2 (10.9–11.5)		
Inpatient	436 (10.3)	66	10.6 (10.0–11.2)		
Education background				258.341	<0.001
College and above	2,111 (49.8)	46	12.7 (12.6–12.8)		
Senior	1,047 (24.7)	71	11.9 (11.7–12.2)		
Junior and below	1,077 (25.4)	201	10.2 (9.9–10.6)		
Transmission route				241.293	<0.001
Homosexual	2,922 (69.0)	117	12.4 (12.3–12.5)		
Heterosexual	1,088 (25.7)	128	11.3 (11.0–11.6)		
Injection drug	155 (3.7)	54	8.7 (7.8–10.8)		
Unknown	70 (1.7)	19	9.4 (8.0–10.8)		
Accepted ART				1,076.444	<0.001
Yes	3,644 (86.0)	98	12.5 (12.4–12.6)		
No	591 (14.0)	220	6.9 (6.2–7.5)		
^*^ Baseline CD4 count level (cell/μL)				48.140	<0.001
<200	1,010 (23.8)	91	10.8 (10.6–11.0)		
200–349	1,091 (25.8)	40	12.3 (12.1–12.6)		
350–499	1,076 (25.4)	43	12.4 (12.1–12.6)		
≥500	918 (21.7)	34	12.2 (11.9–12.5)		

* This variable only included cases that had base CD4 count records.

### Kaplan-Meier survival time of PLHIV

**Figure 2:**
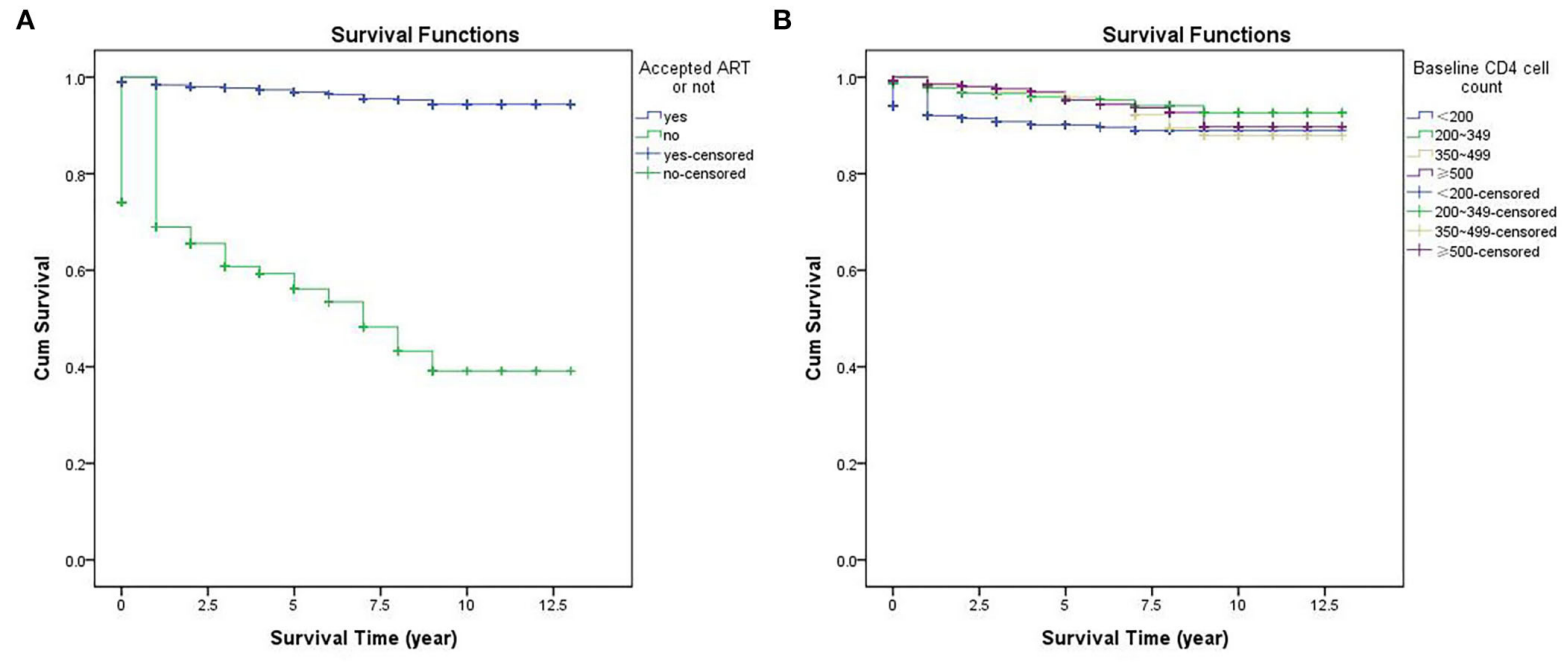
Kaplan Meier survival curves for PLHIV according to different variables: **(A)** accepted ART or not; **(B)** baseline CD4 cell count.

Kaplan-Meier analysis was used to compare the survival time of PLHIV according to accepted ART or not, and different baseline CD4 count levels. The survival curves showed that the cumulative survival rate of PLHIV who accepted ART was remarkably higher than those who unaccepted ART (χ^2^ = 1,076.444, *P* < 0.001). The cumulative survival rate of PLHIV with a baseline CD4 count of <200/μL was lower than those with a baseline CD4 count of ≥200/μL, the difference was statistically significant (χ^2^ = 48.140, *P* < 0.001) (Figures 2A,B).

### Factors associated with mortality of PLHIV

By the log-rank test of Kaplan-Meier, the factors predicting PLHIV survival time were the age of diagnosis, gender, current marital status, education, screening way, transmission route, accepted, ART, and baseline CD4 cell count level (Table 2).

Cox proportional hazards model analysis indicated that ART, baseline CD4 count level, age of diagnosis, education, and screening way remained statistically significantly associated with AIDS-related death. PLHIV who unaccepted ART had a higher risk of death than those who accepted ART, with an *AHR* of 16.2. PLHIV with higher baseline CD4 count had lower death risks than those with <200 cell/μL (*AHR* = 0.4 for CD4 200~349 cell/μL; *AHR* = 0.3 for CD4 350~499 cell/μL; *AHR* = 0.2 for CD4 ≥500 cell/μL). In addition, higher mortality rates were observed among PLHIV who screened for outpatient and inpatient than those from VCT, with *AHR* = 1.56 and 1.64 respectively. Furthermore, the elderly (*AHR* = 9.5 for ≥50 years old; *AHR* = 5.03 for 25~49 years old) and lower educated (*AHR* = 3.4 for junior and below, *AHR* = 1.7 for senior) PLHIV were at higher risk of death. Table 3 shows the above 95% *CI* of *AHR*s.

**Table 3:** Multivariate analysis of the factors associated with mortality among PLHIV in Nanjing.

**Variables**	***P* value**	***AHR* (95% *CI*)**
Age of diagnosis		
15–24		1.0
25–49	<0.001	5.0 (2.0~12.5)
≥50	<0.001	9.5 (3.7~24.1)
Education		
College and above		1.0
Senior	0.020	1.7 (1.1~2.7)
Junior and below	<0.001	3.4 (2.3~5.1)
Screening way		
VCT		1.0
Physical exam	0.737	0.9 (0.5~1.5)
Outpatient	0.012	1.6 (1.1~2.2)
Inpatient	0.018	1.6 (1.1~2.5)
Accepted ART		
Yes		1.0
No	<0.001	16.2 (11.9~22.2)
^*^baseline CD4 count level (cell/μL)		
<200		1.0
200–349	<0.001	0.4 (0.3~0.6)
350–499	<0.001	0.3 (0.2~0.4)
≥500	<0.001	0.2 (0.1~0.3)

* This variable only included cases who had base CD4 count record.

## Discussion

The AIDS-related mortality rate among PLHIV in Nanjing from 2005 to 2018 was lower than the result of a national data study (5.08/100 PYs) (15), as well as Liangshan Prefecture (5.3/100 PYs,) (16) and Guizhou province (7~9/100 PYs) (17). In our study, the 1-year, 5-year, and 10-year survival rates were 95.17%, 91.82%, and 85.42%, respectively. They were higher than Yunnan, Guizhou (southwest China) (16, 17), Henan, and Gansu (northwest China) (18, 19) previously reported results, but lower than the corresponding results of Beijing (the Capital of China) (20). The results showed that the mortality of PLHIV in Nanjing was lower than that of southwest and northwest economically underdeveloped areas of China, but still higher than capital city Beijing.

The unaccepted ART patients' median survival time was only half of those accepted ART, and they had a more than 16-fold risk of adjusted death hazard rate compared with those who accepted ART. This result was consistent with other studies conducted in Beijing (20), Guangdong, (21), and other areas (5), strongly indicating that ART implementation effectively prolonged the survival time of PLHIV. ART reduced HIV replication and infection of new cells and improved the function of the immune system. Moreover, the latest study reported that treating all patients to reduce future population-level HIV transmission could be as efficient as 80% (22). It is worth noting that over half of AIDS-related deaths occurred within the first year of diagnosis, and some even occurred at the same time as HIV infection was confirmed, indicating that most AIDS-related deaths happened before ART or during the initial stage of ART because of the insufficient time to rebuild the immune system. We have seen similar results in other reports, such as Taizhou (Zhejiang Province, China) (23) and Liangshan Prefecture (16).

We found that a higher baseline CD4 count was an independent protective factor, patients with a baseline CD4 count≥200 cell/μL had a lower risk of mortality compared with those baseline CD4 count <200 cell/μL, which was in line with other researchers (5, 24). Since the baseline CD4 count directly reflected immune status, the early onset of infection could facilitate early treatment, thus prolonging the survival time. This may also explain the higher risk of death in outpatient and inpatient than in VCT patients. Outpatients and inpatients are eligible for mandatory HIV testing due to clinical symptoms of immunodeficiency tend to have lower baseline CD4 counts. We have previously confirmed and published these links (25).

Our analysis indicated that age and education were associated with AIDS-related mortality. Elderly PLHIV had more hazards to death than youth. First, elderly PLHIV may have underlying conditions, such as cardiovascular disease and liver or kidney diseases, which would exacerbate opportunity infections and AIDS progression, and subsequently lead to death (26). Second, elderly adults may not consider themselves at risk and be excluded from targeted sexual health information networks (27). Third, elderly patients were less likely to seek medical treatment in the early stage of disease, resulting in delayed diagnosis and higher mortality (5, 21). Last, elderly patients did not receive ART in a timely manner, given the cost, side effects, transportation factors, and lack of company visits.

Our results also showed that patients with higher education had a lower risk of death, which is rare in other studies. Only a previous study reported in Wuhan, China found similar results (28). The reasons may be as follows: on the one hand, highly educated patients are more likely to receive information about the latest epidemic situation, health education, and treatment policies, and can take the initiative to obtain medical services. On the other hand, highly educated patients can get high-paying jobs, and with the support of better economic status, they could obtain better medical resources than those with low education.

There were certain limitations in this study. First, we used observational data from the surveillance information system, and it should be noted that more clinical indicators and other factors were missing. Therefore, further research should be conducted on acceptance or refusal of treatment, the date of initiation of antiretroviral therapy, treatment regimens, and compliance. Second, the cause of death reported in this study may be a misclassification. Although the cause of death was special AIDS-associated syndromes and subjective decision by physicians, death from PLHIV can also be caused by cardiovascular disease, diabetes, and liver disease, as well as by myocardial infarction and metabolic complications as side effects of treatment. Third, we did not consider viral load, which could also be used as a predictor of mortality. Further analysis of viral load may be included (29). Fourth, several patients who followed up in Nanjing may move to other cities during our study period, consequently, we may lose their prognostic information.

Despite these limitations, this is the study to evaluate the survival status of all PLHIV in Nanjing. As another advantage of our study, the large sample size enhanced the reliability of our results.

## Conclusion

Our study described the survival status of PLHIV from 2005 to 2018 in Nanjing. The AIDS-related mortality among PLHIV in Nanjing was relatively low. We observed a higher risk for AIDS-related death among PLHIV who unaccepted ART, whose baseline CD4 cell count was <200 cell/μL, elderly, and lower educated. To reduce the mortality rate further, we should strengthen the publicity of the necessity and importance of accepting ART, and ensure that every case accepts standardized ART as soon as possible. We also need to expand the coverage of HIV detection and early diagnosis of infected cases. Moreover, the publicity and behavioral intervention should focus on the elderly and the lower educated population.

## Data availability statement

The raw data supporting the conclusions of this article will be made available by the authors, without undue reservation.

## Ethics statement

The studies involving human participants were reviewed and approved by Ethics Committee of Nanjing Center for Disease Control and Prevention. Written informed consent from the participants' legal guardian/next of kin was not required to participate in this study in accordance with the national legislation and the institutional requirements.

## Author contributions

SW and XL contributed to data collection. XD and WX contributed to laboratory testing. YX and HS contributed to data analysis. ZZ contributed to study design, data analysis, and manuscript writing. All authors read and approved the final manuscript.

## Funding

This study was supported by the Nanjing Municipal Key Medical Science and Technology Development Project (Grant no. ZKX19050) and the Nanjing Key Medical Specialty Project of Infectious Diseases. The founders had no role in study design, data collection and analysis, the decision to publish, and preparation of the manuscript.

## Conflict of interest

The authors declare that the research was conducted in the absence of any commercial or financial relationships that could be construed as a potential conflict of interest.

## Publisher's note

All claims expressed in this article are solely those of the authors and do not necessarily represent those of their affiliated organizations, or those of the publisher, the editors and the reviewers. Any product that may be evaluated in this article, or claim that may be made by its manufacturer, is not guaranteed or endorsed by the publisher.
